# c-Jun N-Terminal Kinase Inhibitors as Potential Leads for New Therapeutics for Alzheimer’s Diseases

**DOI:** 10.3390/ijms21249677

**Published:** 2020-12-18

**Authors:** Stephanie Cristine Hepp Rehfeldt, Fernanda Majolo, Márcia Inês Goettert, Stefan Laufer

**Affiliations:** 1 Graduate Program in Biotechnology, University of Vale do Taquari (Univates), Lajeado CEP 95914-014, Rio Grande do Sul, Brazil; rehfeldt.stephanie@gmail.com (S.C.H.R.); nandamajolo@gmail.com (F.M.); 2 Brain Institute of Rio Grande do Sul (BraIns), Pontifical Catholic University of Rio Grande do Sul (PUCRS), Porto Alegre CEP 90619-900, Rio Grande do Sul, Brazil; 3 Department of Pharmaceutical/Medicinal Chemistry, Institute of Pharmaceutical Sciences, Faculty of Sciences, University of Tuebingen, D-72076 Tuebingen, Germany

**Keywords:** c-Jun N-terminal kinase (JNK), brain diseases, therapeutic targets, kinase inhibitors

## Abstract

Alzheimer’s Disease (AD) is becoming more prevalent as the population lives longer. For individuals over 60 years of age, the prevalence of AD is estimated at 40.19% across the world. Regarding the cognitive decline caused by the disease, mitogen-activated protein kinases (MAPK) pathways such as the c-Jun N-terminal kinase (JNK) pathway are involved in the progressive loss of neurons and synapses, brain atrophy, and augmentation of the brain ventricles, being activated by synaptic dysfunction, oxidative stress, and excitotoxicity. Nowadays, AD symptoms are manageable, but the disease itself remains incurable, thus the inhibition of JNK3 has been explored as a possible therapeutic target, considering that JNK is best known for its involvement in propagating pro-apoptotic signals. This review aims to present biological aspects of JNK, focusing on JNK3 and how it relates to AD. It was also explored the recent development of inhibitors that could be used in AD treatment since several drugs/compounds in phase III clinical trials failed. General aspects of the MAPK family, therapeutic targets, and experimental treatment in models are described and discussed throughout this review.

## 1. Introduction

In 1906, Dr. Alois Alzheimer described for the first time an unusual disease of the cerebral cortex. According to the German neurologist, the patient whose brain he examined was a woman who had been suffering from memory loss, disorientation in time and space, hallucinations, and language dysfunction. She eventually died at 55 years of age. During a post-mortem examination of the patient’s brain, he noted that the cortex was thinner than average, and he also observed the presence of abnormalities such as plaques and tangles in and outside the brain cells [1,2]. Today, the ‘unusual disease’ is known as Alzheimer’s Disease (AD), and, as the population lives longer, diseases such as AD are becoming more prevalent. Researchers believe the overall prevalence of AD in individuals over 60 years of age is 40.19% across the world [3]. Five years ago, the annual World Alzheimer Report of 2015 pointed out that the percentage of individuals over 60 years old diagnosed with AD corresponded to 12.2% of the world population at that time. The same report projected an increase in that percentage to 16.3% in 2030 and 21.2% in 2050, anticipating that the number of cases would almost double every 20 years [4,5].

It has been 114 years since the first official AD diagnosis and considerable progress has been made, but some complex aspects remain unclear. AD is a multifactorial disease as it appears to be a result from genomic, epigenetic, interatomic, and environmental aspects interacting in different ways, resulting in highly heterogeneous phenotypes. Clinically, AD is divided into ‘late-onset’ or ‘sporadic’ AD (LOAD)—which is the most common form of AD—and ‘early-onset’ or ‘familiar’ AD (EOAD), which accounts for 1-5% of all AD cases and is caused by dominantly inherited mutations in genes including *APP*, *PSEN1*, *PSEN2*, and *APOE* [6]. Genetic evidence indicates that heritability to LOAD is 58-79%, while for EOAD is thought to be over 90%. Recently, over 50 *loci* were identified and linked to LOAD reinforcing the role of multiple pathways and cellular events, such as immunity, endocytosis, cholesterol transport, ubiquitination, amyloid-β, and τ (tau) processing [7].

There are several descriptive hypotheses regarding the causes of AD including the amyloid hypothesis, τ propagation hypothesis, cholinergic hypothesis, mitochondrial hypothesis, inflammatory hypothesis, and others. The most well-known hypothesis is the amyloidogenic one since it describes some classical AD hallmarks [8]. One of these hallmarks is originated from abnormal processing of amyloid precursor protein (APP), a single-pass transmembrane protein that is normally present in neurons and cleaved by several secretases. In AD, APP follows the amyloidogenic pathway where it is cleaved by β-site APP-cleaving enzyme 1 (BACE1) to originate amyloidogenic C-terminal derivatives (sAPPβ) [9], which are cleaved by γ-secretase, generating APP intercellular domain (AICD) and β-amyloid peptides (Aβ), including Aβ42. These toxic oligomers can accumulate in neurites and disrupt the synaptic function. It is believed that disconnected terminals create neurites around Aβ42 deposits, originating the neuritic plaques (NP) [10]. A second common finding in AD are intracellular neurofibrillary tangles (NFTs) composed of poorly soluble hyperphosphorylated τ protein. τ protein is a microtubule-associated protein normally synthesized by neuronal cell bodies and transported to axons, where it interacts with tubulin to assure microtubule stability. If there are perturbations in the cytoskeleton, it can compromise both the function and viability of the neurons. Even after cell death, it is still possible to observe the extracellular NFTs left behind [11,12]. It was believed that NP and NFTs acted independently, but recent evidence points to a complex relationship where they act synergistically towards neurodegeneration [13]. High levels of Aβ aggregates and NFTs distributed mainly throughout the hippocampus lead to critical events. Those molecular and morphological abnormalities are the hallmarks of brain injury observed in dementia and are likely to develop over a period of at least decades before the symptomatic phase. A study found significantly higher levels of total τ (T-τ) in cerebrospinal fluid 34 years before the symptomatic onset of AD, while changes in cognitive aspects were detected 10 to 15 years prior to the symptomatic phase [14]. In addition, the distinct pattern of plasma levels of τ phosphorylated at Thr217 could discriminate AD from other neurodegenerative diseases and are therefore gaining attention as a potential biomarker for AD [15,16,17].

While Aβ is a necessary feature to diagnose AD in a patient, aggregates are not sufficient to cause cognitive decline [18,19]. In this case, synaptic dysfunction, oxidative stress, and excitotoxicity, which induces the activation of mitogen-activated protein kinases (MAPK) pathways such as the JNK (c-Jun N-terminal kinase) pathway, are also necessary to provoke progressive loss of neurons and synapses, brain atrophy, and augmentation of brain ventricles [20,21,22,23,24,25]. Considering that JNK is best known for its involvement in propagating pro-apoptotic signals via extrinsic and intrinsic pathways [26,27,28], the inhibition of JNK3 has been explored as a possible therapeutic target. Nowadays, AD symptoms are manageable, but the disease itself remains incurable. According to the U.S. Food and Drug Administration (FDA) and the Alzheimer’s Association, no drug available today for AD is able to slow down the progression of the disease nor stop the neuron cells from dying, which makes AD fatal in all cases. On the other hand, there are five FDA-approved drugs to manage AD nowadays: Donepezil, galantamine, memantine, rivastigmine, and an association of memantine and donepezil [29]. Tacrine used to be a sixth drug available for the same purpose, but it was discontinued in 2013, after having gained approval 20 years earlier, because of increased hepatotoxicity [30]. When considering the failure of several drugs/compounds in phase III clinical trials that focused primarily on the amyloid hypothesis [31], it becomes clearer that new targets should be explored. This review presents the role of JNK3 in AD pathogenesis and explores the recent development of inhibitors that could be used in AD treatment. In this sense, we first describe the general aspects of the MAPK family. Next, we discuss the biological aspects of JNK, focusing on JNK3 and how it relates to AD. Subsequently, we explore therapeutic targets and experimental treatment in models.

## 2. General Aspects of Mitogen-Activated Protein Kinases (MAPKs) Family

Kinases are considered the largest protein family in the human proteome, and approximately 2% of eukaryotic genes encode kinase superfamily members [32]. These enzymes catalyze the transference of the γ-phosphate from ATP to serine, threonine, or tyrosine amino acid residues of a downstream protein substrate, creating a communication cascade that is fundamental to eukaryotic cells. Over 500 kinases were identified in humans. The so-called ‘human kinome’ is divided into typical kinases and atypical kinases. The 478 typical kinases possess a well-defined architecture that is similar across all members, while the 40 atypical kinases (the ‘dark kinome’) are poorly understood [33]. Most typical kinases are dually phosphorylated at serine and threonine residues and, as a result, are called Ser/Thr kinases. Kinases are clustered according to similarities at the kinase domain, which results in different kinase groups/families. According to evolutionary conservation data, the CMGC kinase group is an ancient group made up by nine highly conserved families found in most eukaryotes [34]. This group was named after the initials of some of its key members: cyclin-dependent kinase (CDK), MAPK, glycogen synthase kinase (GSK), and CDK-like kinases (CLK).

**Figure 1:**
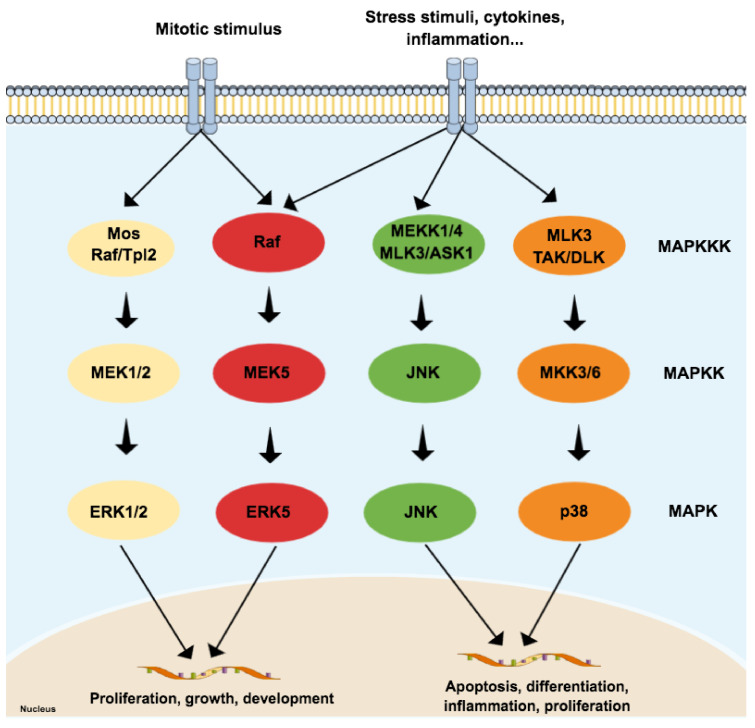
Schematic representation of the typical mitogen-activated protein kinases (MAPK) pathway, composed of three levels (three-tier module) and four subfamilies: extracellular signal-regulated kinases (ERK) 1/2, p38, c-Jun N-terminal kinase (JNK), and ERK5. Each subfamily regulates different phenotypes. While the canonical ERK subfamily (ERK1/2) responds to mitotic stimuli and controls cellular differentiation and proliferation, JNK and p38 are activated by stressor stimuli and are involved in apoptosis. Since the ERK5 subfamily responds to both mitotic and stressor stimuli, being associated with cell survival, it is presented in a special subfamily.

The MAPK family is considered the main propagator of extracellular signals from the cell membrane to the nucleus as they catalyze the transfer of the γ-phosphate from ATP to serine or threonine residues of various substrates [35], including transcription factors, which regulate the expression of specific sets of genes, and thus mediate a specific response according to the stimulus received by cells [36,37,38,39]. Among the MAPK family, fourteen members share both structures and biochemical properties. Depending on those characteristics, each member is grouped in one of the seven different MAPK subfamilies. The extracellular signal-regulated kinases 1/2 (ERK1/2), ERK5, JNK, and p38 subfamilies can be activated by dual phosphorylation of threonine and tyrosine residues (known as ‘tripeptide motif’ or ‘Thr-X-Tyr motif’) at the activation loop site (also called ‘A-loop’) and activate the pathway by propagating the phosphorylation in downstream kinases. Those four subfamilies follow a classical three-tiered signaling pathway that is highly conserved in eukaryotic organisms, where each phosphorylation of a MAPK is carried out by specific upstream kinases. Usually, MAPKKK (MAP3K) receives a variety of inputs and transmits them to downstream kinases MAPKK (MAP2K), which activates MAPK (Figure 1) [40,41,42]. On the other hand, ERK3/4, ERK7/8, and NLK (nemo-like kinase) compose the ‘atypical’ MAPK subfamilies that do not follow the same dual-phosphorylation and three-tiered module pattern. However, since the focus of this paper is on ‘typical’ MAPK—specifically in JNK—the ‘atypical’ MAPK will not be reviewed in detail and will be referred to as appropriate. A detailed review of their dynamics can be checked elsewhere [40,41].

MAP3K activates more than one MAP2K, and MAP2K can phosphorylate more than one MAPK. The cross-talking is important to signal integration and coordination but may also represent an obstacle in therapy since it is likely to be involved in drug resistance in cancer, for example [43]. Although the MAPK pathways have several interconnections that sometimes hinder the separation of these pathways, the JNK and p38 pathways are both activated by cellular stressors, such as cytokines for example, and are frequently associated with cellular death and inflammation [36,39,44]. On the other hand, both JNK and p38 can mediate anti-apoptotic events as well and do not always work ‘as a team’, since recent evidence suggests negative regulation of p38 over JNK in some cellular contexts [45].

### 2.1. JNK3 (and p38), Apoptosis and AD: The Perfect Storm

#### 2.1.1. JNK Participates in Both Intrinsic and Extrinsic Pathways of Apoptosis

**Figure 2:**
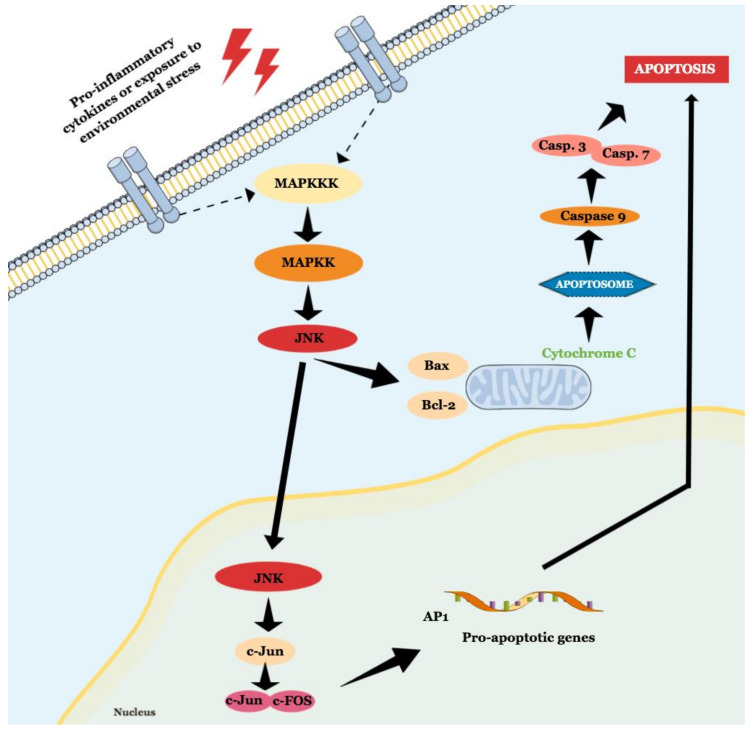
Schematic representation of JNK role in intrinsic and extrinsic apoptosis.

JNK is a MAPK activated by pro-inflammatory cytokines or exposure to environmental stress and is best known for its role in programed cell death [46]. The term ‘apoptosis’ was suggested in 1972, and it describes a regulated cell death process that requires a complex molecular program of self-destruction where cells retain plasma integrity and some level of metabolic activity until the outcome [47]. Apoptotic processes are divided into extrinsic and intrinsic pathways, and a detailed review of this topic is available elsewhere [47,48]. Here we will discuss general aspects that involve the role of JNK in apoptosis, as illustrated in Figure 2.

The intrinsic pathway is activated by extracellular or intracellular perturbations usually found in AD, such as oxidative stress and microtubular alterations caused by NTFs [12,49]. This pathway is controlled by members of the Bcl-2 family, which is divided into three groups known as pro-apoptotic pore-formers (Bax, Bak, and Bok), pro-apoptotic BH3-only proteins (Bad, Bid, Bik, Bmf, Hrk, Noxa, Puma, etc), and anti-apoptotic proteins (Bcl-2, Bcl-XL, Bcl-W, Mcl-1, Bfl-1) [50]. The Bcl-2 family is under the control of JNK and p38 [45], and a significant part of them can transit between cytosol and organelles. In response to a deleterious stimulus, the JNK-mediated phosphorylation of 14-3-3 protein at the Ser184/186 site induces the translocation of pro-apoptotic proteins (Bax and Bad) from the cytoplasm to the mitochondria. However, it was reported that JNK can directly phosphorylate Bad at Ser128, Bim at Ser65, and Bid at Thr59, inducing the pro-apoptotic activity, and inhibiting anti-apoptotic proteins, since Bad, Bim, and Bmf inhibit Bcl-2 antiapoptotic effect [48,49,51]. JNK also phosphorylates Bcl-2 and Mcl-1, blocking their anti-apoptotic activity. This pathway is also known as the ‘mitochondrial pathway’ since the relocation of pro-apoptotic proteins causes mitochondrial outer membrane permeabilization (MOMP), allowing the cytosolic release of pro-apoptogenic factors that normally reside in the mitochondrial intermembrane space, such as cytochrome c and Smac/DIABLO. Cytochrome c then associates with Apaf-1, pro-caspase 9 (CASP9), (and possibly other proteins) to form an apoptosome, which activates CASP9. When activated, CASP9 catalyzes the proteolytic activation of CASP3 and CASP7 (known as ‘executioner caspases’), which handle cell demolition during intrinsic and extrinsic apoptosis pathways. In this sense, both pathways can induce caspase activation that causes the morphological and biochemical features commonly seen during apoptosis, including DNA fragmentation and phosphatidylserine exposure [52]. However, both the pathways usually operate independently, since Bid, a pro-apoptotic Bcl-2 family member, can be cleaved by CASP8, promoting cytochrome c release [49].

The extrinsic pathway initiates when perturbations on the extracellular environment are detected by cell-surface death receptors (or ‘dependence receptors’) and therefore is also known as ‘death-receptor pathway’. Death receptors include Fas (known as CD95 or APO-1), tumor necrosis factor receptor (TNFR), TNF-related apoptosis-inducing ligand (TRAIL), and others [53]. The dual role of Fas in both apoptotic and anti-apoptotic signaling is under debate. In animal models, repetitive and coordinated activation of Fas improved spatial memory and increased adult neurogenesis in the hippocampus, indicating that Fas could be involved in neuroprotection rather than neurodegeneration in mild cognitive impairment (MCI) [53,54,55,56]. However, depending on the cellular context, these receptors activate CASP8 and propagate the apoptotic signal by direct cleavage of CASP3 and Bid, converging to the mitochondrial pathway. On the other hand, the extrinsic pathway can be activated according to the stimulus perceived by MAP3K, such as transforming growth factor-β-activated kinase 1 (TAK1), mammalian MAP/ERK kinase kinase 1 (MEKK1), MEKK4, apoptosis signal-regulating kinase 1 (ASK1), and mixed-lineage kinase (MLK), by GTPases like Ras (Raf) or GTPases of the Rho family (Cdc42-mediated MLK3 activation and RhoA-mediated MEKK1 activation) or, further, phosphorylated by a MAP4K, such as p21-activated kinase (PAK), germinal center kinase (GCK), and homeodomain-interacting protein kinase (HPK), involved in the control of MAP3K interaction with other proteins. This diversity in MAP3K regulation allows the activation of MAPK in response to many stimuli and one of the most complex pathways since it involves the recruitment of a large number of MAP3K [23,26,44,57,58,59,60,61,62,63,64,65]. MAP3Ks activate the most important MAP2K substrates in the JNK pathway, such as MKK4 and MKK7. Next, JNK is dually phosphorylated on the Thr-X-Tyr motif on Thr-221 by MKK7 and Tyr-223 by MKK4, preferentially. However, for JNK activation, an interaction between scaffold proteins JNK-interacting protein (JIP) 1-3 and the ‘ASK1-MKK4/7-JNK’ complex still provides a structural modification in this complex for the correct phosphorylation of JNK, which results in Bid cleavage. Besides JIP 1-3, other proteins such as POSH (plenty of SH3s) are also critical for complex anchoring since they appear to facilitate an interaction between GTP-Rac1 and subsequent proteins [23,26,44,59,61,66,67,68,69,70,71]. Then, JNK phosphorylates transcription factors, which induces the expression of pro-apoptotic proteins and decreases the expression of anti-apoptotic proteins. The major JNK target is the transcription factor AP-1, which is a complex formed by members of Jun, Fos, ATF, and MAF protein families. JNK phosphorylates ATF2 at the NH_2_-terminal activation domain on Thr69 and Thr71 residues, increasing ATF2 transcriptional activity. JNK phosphorylates the δ-domain on the NH_2_-terminal region of c-Jun on Ser63 and Ser73, which increases c-Jun transcriptional activity. JunD is a poor substrate, while JunB binds to JNK (2-fold less when compared to c-Jun) but is not a substrate. The δ-domain in the NH_2_-terminal region (present in c-Jun) is required for the phosphorylation but poorly conserved within the other two members (JunD and JunB) of the Jun family of transcription factors. Deletion of the δ-domain domain blocks the phosphorylation by JNK, indicating it could be a potential target. Besides the fact that the combination of the components of AP-1 might vary, each isoform displays differences in binding affinity to the JNK substrates [23,39,63,69,72,73]. While binding of JNK2β1 and JNK2β2 to ATF2 is 2-fold greater than the binding to c-Jun, binding of JNK2α1 and JNK2α2 to c-Jun is 2-fold greater than ATF2 [74]. Another substrate for JNK is p53, another protein responsible for increasing the expression of pro-apoptotic proteins in stress-induced cell death. To induce this death signal, JNK phosphorylates p53 at Thr81, allowing p53 to form a dimer with p73. This p53-p73 complex induces the expression of pro-apoptotic genes such as *puma* and *bax*. On the other hand, p53 can trigger the MOMP as well in a transcription-independent manner by activating pro-apoptotic Bcl-2 proteins (Bax or Bak) or by inactivating anti-apoptotic Bcl-2 proteins (Bcl-2 and Bcl-X1). Other transcription factors were reported as JNK substrates and could also induce apoptosis in different stress situations, indicating that JNK pathway activation is context-specific and cell type-specific [45].

#### 2.1.2. JNK Isoforms: Are They All the Same Thing?

The human genome contains three genes known as *JNK1* (*MAPK8*), *JNK2* (*MAPK9*), and *JNK3* (*MAPK10*), which encode several JNK isoforms via alternative splicing of mRNA. In 1994, JNK1 isoform was isolated from a fetal brain cDNA library [45], while a second isoform, JNK2, was isolated from HeLa [75] and Jurkat [76] cDNA libraries. In 1996, while screening adult brain cDNA libraries, a group confirmed the two previously identified isoforms and found eight new sequences that corresponded to novel JNK isoforms [74]. For both JNK1 and JNK2, four isoforms were identified for each enzyme, a total of eight isoforms. These alternative sequences show differences in COOH-termini and protein kinases subdomains IX and X. For JNK3, two isoforms with different COOH-termini but the same IX and X subdomains were identified as well. The major difference between JNK3 and the other JNK is an extended NH_2_-terminal region fused in-frame to the conserved methionine residue that serves as the NH_2_-terminus of the other JNK. The isoforms are listed in the following table (Table 1):

The expression of multiple JNK isoforms is fundamental to generate tissue-specific responses to the activation of JNK. Although all three isoforms and their upstream activators are indispensable to neurodevelopment; several human tissues express both the JNK1 and JNK2, while JNK3 expression is limited to a specific subset of neurons in the nervous system. Somehow JNK3 appears to be associated with multiple neuropsychiatric conditions, such as epileptic encephalopathy [79] and anxiety induced by hepatic encephalopathy [80]. It was reported in the literature that JNK3 knockout in mice (*JNK3^-/-^*) leads to significant SNC-associated defects and embryonic death [81]. A weak expression was found in other tissues in both pathological and physiological conditions [82].

#### 2.1.3. Brain Regions Normally Affected in AD Are the Same Regions Where JNK3 Expression Was Identified. Is It a Coincidence?

One of the first pieces of evidence that fed the hypothesis associating JNK3 and AD was described 15 years ago [83] when it was demonstrated that the expression of JNK3 is co-located with ALZ-50 antigen in brains in AD hippocampal sections. This co-occurrence indicates the presence of both JNK3 expression and abnormally phosphorylated τ protein, which is a precursor for the formation of NTF [11,12,84]. At that time, researchers showed for the first time the presence of JNK3 in specific tissues. Northern blot assay performed using mRNA from heart, placenta, lung, liver, skeletal muscle, kidney, pancreas, and testis identified a weak hybridization in kidney and testis tissues, while other tissues showed no hybridization. In addition, analysis of JNK3 mRNA by both Northern blot and in situ hybridization in post-mortem brain samples of neurotypical individuals ranging from 35 to 56 years old found JNK3 expression in a particular subset of pyramidal neurons in CA1, CA4, and subiculum regions of the hippocampus and layers III and V of Brodmann’s areas 4, 10, and 17 of the neocortex. JNK3 was also identified in the cerebellum, striatum, and brain stem, and a weak signal was detected in the spinal cord as well. There was no expression of JNK3 found in white matter that lacks neuronal cell bodies [83].

The clinical presentation of AD indicates that neuronal death and dysfunction affect specific brain regions that are critical to memory functioning, learning, and cognitive performance [84]. As discussed on the previous topics, a perturbation in these brain networks occurs because of different aspects including cytoskeletal abnormalities in neurons due to NFT and NP, the presence of Aβ plaques in regions responsible for receiving inputs from other regions causing synaptic interruption, reductions of neurotransmitters, loss of neurons, and local glial inflammatory reactions usually associated with plaques. Those regions include the basal forebrain, cholinergic system, hippocampus, entorhinal cortex, limbic cortex, and neocortex, and different researchers demonstrated that AD patients showed an anatomic alteration in their brains via MRI such as hippocampal atrophy, with subsequent temporal atrophy and ventricular expansion [85]. Those relationships suggest some possible correlations of the architectonic distribution of neurodegenerative damage and brain circuits involved in the control of certain social and emotional behaviors and are summarized in Table A1.

By mapping 39 cortical areas of 11 brain samples from AD patients, one study revealed that higher levels of NFT were found in the limbic penallocortex (area 28), subiculum and CA1 zones of the hippocampus (area 51), basal nucleus of the amygdala (areas 11, 12, and 24), anterior insula (areas 38 and 35), non-primary association cortex (areas 32, 46, 40, and 39), posterior parahippocampal cortex (areas 37 and 36), primary sensory association cortex (areas 7, 18, 19, 22, 21, and 20), agranular cortex (areas 44, 45, 8, 6, and 4), and primary sensory cortex (areas 41 and 42). The distribution of NFT appeared to be more selective to limbic and temporal lobes than frontal, parietal, or occipital lobes, while there were more neuritic plaques (NP) in temporal and occipital lobes [84]. Still, when considering atrophy in well-defined brain structures and AD symptoms, areas with higher expression of JNK3 are also in evidence. Research found regions of decreased gray matter volume associated with neuropsychiatric behaviors in patients with mild AD. Delusion symptoms were associated with decreased gray matter density in the left frontal lobe (medial frontal gyrus/area II and inferior frontal gyrus/area 45), in the right frontoparietal cortex (inferior frontal gyrus/area 45 and inferior parietal lobule/area 40) and the left claustrum. Apathy, which is often seen in early AD and may be present even before noticeable memory deficits, was associated with gray matter density loss in the anterior cingulate (area 24) and frontal cortex bilaterally (inferior frontal gyrus/area 47, middle frontal gyrus/area 9, superior frontal gyrus/area 10) the head of the left caudate nucleus and in bilateral putamen (nucleus lentiform), and agitation was associated with decreased gray matter values in the left insula (area 13), and the anterior cingulate cortex bilaterally (area 24). Those findings suggest that AD symptoms are associated with neurodegeneration in specific neural networks supporting personal memory, reality monitoring, processing of reward, interoceptive sensations, and subjective emotional experience [86].

The expression profile of JNK3 suggests it is expressed by cells in specific brain regions commonly affected in AD patients. More recently, studies have emphasized the role of JNK3 in neurodegenerative diseases like AD. In this sense, studies on post-mortem brain samples have shown a greater expression of phosphorylated JNK3 in AD patients besides the presence of Aβ [87,88,89]. Further studies have identified that JNK3 is highly expressed and activated in brain tissue and cerebrospinal fluid in patients with AD, besides being statistically correlated with the level of cognitive decline [90,91]. In fact, in 2012, one study showed that the activation of JNK3 is essential for the pathophysiology of AD by maintaining positive feedback on Aβ42 production [92]. The Aβ42 activates AMPK, inhibiting the mTOR route, leading to oxidative stress, which activates JNK3. Once activated, JNK3 promotes the processing of APP by phosphorylating the protein at position T668P, which induces the internalization of APP, facilitating its cleavage/phosphorylation directly, favoring the production of Aβ42, restarting the cycle. From this study, JNK3 was shown to be the main kinase promoter of APP phosphorylation in T668, since *JNK3^-/-^* knockout mice showed a dramatic reduction in Aβ42 levels, and higher numbers of neuronal cells, and better cognitive function.

At the epigenetic level, unique patterns of methylation in CpG and CpH sites of enhancers were observed, suggesting that epigenetic control of these enhancers is involved in AD and neuronal dysfunction. Even though the CpH methylation decreases with age, it was accelerated in AD. In samples of the prefrontal cortex of individuals diagnosed with AD, JNK3 (*MAPK10*) showed enhancer hypomethylation [93]. This finding, therefore, corroborates with other researchers that found overexpression of JNK3 in specific brain areas and reinforces the role of JNK3 in inducing some AD hallmarks. In the middle temporal gyrus, it was found that JNK3 (*MAPK10*), Bcl-2 family members (Bid, BAK1), and multiple caspases (CASP8, CASP3, CASP7) for example, were also hypomethylated in patients with AD, suggesting an upregulation of apoptotic pathways in AD neurons [94]. Human-induced pluripotent stem cell-derived neurons carrying isogenic apoE3/3 transplanted to apoE4/4 knock-in mouse hippocampus showed dysregulation of many pathways involved in cell stress, such as apoptosis. It was revealed that JNK3 (*MAPK10*) was the gene implicated in the largest number of dysregulated pathways [95].

The overall importance of JNK signaling in brain development results from the multitude of basal functions such as regulating region-specific neuronal death or migration and neuronal polarity, neuronal regeneration, learning, and memory, for example [44]. On the other hand, JNKs are expressed in microglia, astrocytes, and oligodendrocytes as well as in neurons [96,97], and despite having different roles, microglia and neurons ‘communicate’ often, and one way to do so is via JNK signaling axis [98] showing its fundamental role in both pathological events such as neuroinflammation and physiological processes like regeneration.

#### 2.1.4. JNK and p38 Working Together towards Chaos

Despite glial cells having been reported to induce deregulation in mitochondria and endoplasmic reticulum dysfunction resulting in bioenergetic and Ca^2+^ homeostasis disruption [99], neuroinflammation is critical for AD since it appears to modulate the disease progression [100,101]. The integration of the immune system and the central nervous system was recently reviewed [102]. In AD, the neuroinflammation relies on innate immune responses mediated by microglia [103]. The microglia, a subtype of neuroglial cells, comprise resident immunocompetent and phagocytic cells originated from yolk sac-derived erythro-myeloid progenitors that gain CNS-surveilling properties during early fetal development, and are not replaced by circulating bone marrow-derived cells throughout their lifespan [104,105], and represent 8%-20% of all CNS cells [106,107,108]. On the other hand, they share some phenotypic traits and innate immunological functions with peripheral macrophages since they express major histocompatibility complex (MHC) antigens, and T and B cell markers, but differ from other tissue macrophages due to their tight regulation by the CNS microenvironment [109]. Among other functions, the key role of the microglia consists of the regulation of inflammation, synaptic connectivity, programed cell death, wiring and circuitry formation, phagocytosis of cell debris, and synaptic pruning and sculpting of postnatal neural circuits [110]. Research identified a correlation of specific expression patterns in microglia from the brain cortex and AD risk variations, indicating that neuroinflammation may have a more important role in AD than in other neuropsychiatric diseases [99]. It was revealed that inflammatory pathways are also linked to rapid cognitive decline in the mild cognitive impairment stage of AD [18]. Thus, a link between some AD hallmarks and neuroinflammation continues to grow stronger. It was reported that microglia from animals carrying the ε4 allele of the *apoE* gene are deficient in clearing Aβ aggregates [95]. Despite the major function of apoE being associated with cholesterol transportation, it is usually related to increasing Aβ clearance by promoting migration and activating phagocytosis in microglia, and mutations are associated with EOAD [103]. However, it was suggested that Presenilin1 also modulates Aβ clearance [111].

In the pre-clinical stages of AD, the microglia play a protective role by phagocyting and depredating toxic Aβ aggregates. On the other hand, as the disease progresses, the Aβ clearance by microglia appears to decrease due to overstimulation [100,101,108,112]. In other words, after some point, microglia cells appear to gradually lose their phagocytic phenotype. In vivo AD models corroborate this hypothesis, since it was found that microglia decrease the expression of their Aβ-binding receptors and Aβ-degrading enzymes while maintaining the ability to produce pro-inflammatory cytokines [113]. For a time, it was believed that the microglia under physiological conditions (so-called ‘resting microglia’) could switch into a reactive morphology, being called ‘activated microglia’ through activation of macrophage polarization in which it could acquire an M1 or an M2 phenotype. The M1 phenotype is associated with pro-inflammatory induction, similar to activated peripheral macrophages and Th cells, while the M2 phenotype is associated with an anti-inflammatory state [110,114]. Recently, this categorization has been under discussion because it is difficult to fully characterize the ‘pro-inflammatory’ phenotype of microglia in neurological diseases, and the classification appears to be too restrictive, which hinders research progress [115]. For further information about the role of microglia in neurodegenerative diseases, please check the following references [116,117].

Despite the controversy, it is widely accepted that in the past two decades, neuroinflammation has been considered an important component of the disease pathogenesis. As mentioned above, microglia may contribute to neurodegeneration effects since it reacts to Aβ, for example. One mechanism of Aβ clearance in the brain is the uptake and degradation of those aggregates by the microglia-mediated by Toll-like receptors (TLRs) TLR2 and TLR4, for example. After a stimulus, the microglia produce several inflammatory mediators, such as IL-1β, IL-6, TNF-α, prostaglandin E2 (PGE2), nitric oxide (NO), brain-derived neurotrophic factor (BDNF), which can activate the JNK pathway. The major contribution of JNK to neuroinflammation is via its transcription factor, AP-1, which regulates pro-inflammatory genes such as *COX2*, *NOS2*, *TNF-α*, *CCL2*, and *VCAM-1* [74]. The CCL2 level in cerebrospinal fluid (CSF) is comparable to the hippocampal volume in predicting a rapid cognitive decline in MCI, a preclinical phase of AD [18]. ATF2, another JNK substrate, is also associated with pro-inflammatory genes. The activation of JNK contributes to AD pathophysiology through pro-apoptotic and pro-inflammatory effects.

On the other hand, studies indicate that microglia may contribute to the pathology of AD through the production of IL-1, activation of neuronal p38-MAPK, and synaptic and cytoskeletal alterations [107,108], which manages the aggregation of NTFs [8,19,22,36,118], which confirms their participation in AD, and thus become an important therapeutic target. p38 is a 38 kD polypeptide MAPK found in 4 isoforms (α, β, γ, δ) and it is strongly activated by environmental stresses and inflammatory cytokines [119,120]. Similar to JNK, phosphorylated p38 was found especially in the CA2 and the subiculum, but at the CA1 in the hippocampus as well [121], and near NP and NTF in *post-mortem* brains of AD patients [35,122,123]. In response to Aβ, NTF, or oxidative stress, also a common feature in AD brains, JNK and p38 induce NF-kβ, synaptic excitotoxicity, and neuroinflammation [123,124,125,126,127]. Corroborating these findings, it was demonstrated that NJK14047 (selective p38α/β inhibitor) reduced NOS, COX-2, TNF-α, and IL-1β in vitro, decreased microglia activation in vivo [128], and improved cognitive functions in an AD mouse model [129]. This could be explained by the fact that p38α expression stimulates BACE1 and, therefore, the Aβ generation, but also induces dysfunctional autophagy in neurons [129]. Evidence suggests that patients with neurodegenerative diseases such as AD might also benefit from p38 MAPK inhibitors [130]. Besides, p38 might modulate (at some point) psychiatric symptoms observed in AD such as depression [131]. The prevalence of major depression in demented patients is 32% [132], in AD patients is over 20% [131], while the lifetime prevalence of depression is 10.8% [133]. Evidence showed that IL-1β and TNF-α increased the serotonin transporter (SERT) activity (and therefore decreased synaptic availability of serotonin) via p38 activation in vitro and in vivo [134,135]. LPS-treated mice showed increased SERT activity and depressive-like behavior, but when the animals were treated with SB203580, increased serotonin uptake and depressive symptoms were no longer observed [135].

Other studies showed that neuronal deletion of p38α had neither impact on the hippocampal and cortical development nor on learning and memory skills, motor coordination, and muscle function. On the other hand, the animals showed increased anxiety behavior and higher levels of JNK activation on the CA1, CA3, and dentate gyrus. Inhibition of JNK using SP600125 or D-JNKi, however, decreased JNK activation in vitro and ex-vivo and reverted the anxiety-like behavior induced by deletion of p38α in vivo. This suggests that the specific activation of the p38α isoform is necessary to control behavioral states related to anxiety through JNK inhibition in CNS since other isoforms did not decrease JNK levels in both in vivo and in vitro models [136]. It opens an interesting discussion because the selective inhibition of p38 could induce anxiety symptoms principally needs to be taken into account when dealing with elderly individuals.

#### 2.1.5. JNK Structure

**Figure 3:**
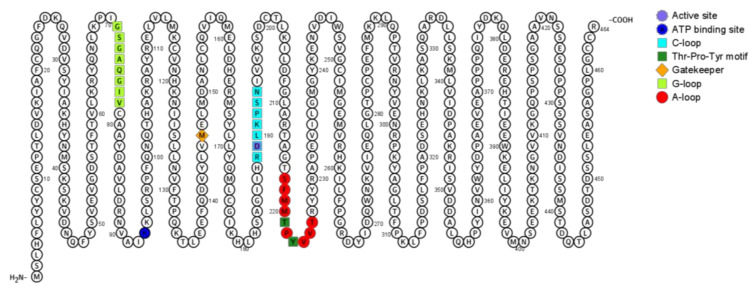
Two-dimensional structure of JNK3. Key structure elements are colored as shown in legend. Figure generated with Protter.

Similar to other kinases, JNK presents 11 subdomains (I-XI) in a conserved arrangement of two lobes, the C-terminal lobe and the N-terminal lobe that are connected through a catalytic site (the ATP-binding site) and signature sequences that contribute to proper stabilization [137,138]. In the N-lobe, at the subdomain I, between the β1 and β2-strands there is a glycine-rich sequence Gly-X-Gly-X-X-Gly-X-X (also called ‘G-loop’, residues Gly71 to Val78) that comprises the ATP-binding site and is followed by a valine residue at the β2-strand that makes a hydrophobic contact with the adenine of ATP. The subdomain II contains Lys43 at the β3-strand that forms a salt bridge with a glutamate residue from subdomain III. So, the third subdomain is composed of a αC-helix and contains Glu111 that interacts with Lys43 (subdomain II) in the active conformation, as the salt bridge couples with ATP. Subdomain IV contains a β4-strand, which contributes to the structure of the N-lobe, while subdomain V contains a hydrophobic β5-strand in the N-lobe and an αD-helix in the C-lobe. The sequence between β5-strand and αD-helix links the N-lobe and the C-lobe. At the subdomain V, there is a gatekeeper residue (Met146) that is positioned deep inside the ATP-binding pocket and is between two hydrophobic regions [139]. This residue takes part in the protein conformation structure and determines the size of the binding pocket. The subdomain VIa contains the αE-helix that parallels the αF-helix from subdomain IX. In the C-lobe, the subdomain VIb influences catalytic reactions as it contains the catalytic loop (‘C-loop’) with a conserved HDR motif (His187-Arg188-Asp189). Asp189 forms a hydrogen bond with Tyr223 and is the catalytic base that accepts the hydrogen removed from the hydroxyl group being phosphorylated. Subdomain VII contains the activation segment, which starts with a DFG motif (Asp207-Phe208-Gly209). This motif is particularly important to activate the protein. When Phe208 is inside the hydrophobic pocket created by residues from the C lobe and N lobe, the conformation is called ‘DFG-in’. On the other hand, if Phe208 is outside the hydrophobic pocket (called ‘DFG-out’ conformation) the enzyme is inactivated as Asp207 can no longer properly orient Mg^2+^ to the active site, and as a result, the transference of phosphate from ATP to the substrate is disrupted [140]. Subdomain VIII contains the threonine and tyrosine residues whose phosphorylation activates JNK (called ‘TYP motif’, Thr221-Pro222-Tyr223), the ‘APE motif’ (Ala231-Pro232-Glu233), and the p+1 loop (Arg227-Arg230), which is between TYP and APE motifs. The region between the DFG motif (subdomain VII) and APE motif (subdomain VIII) is referred to as the activation loop (A-loop, Ser217-Thr226). Once Thr221 and Tyr223 are phosphorylated, the protein changes its conformation so that the activation loop is oriented to render the active site cleft accessible; DFG and HDR motifs are properly positioned for catalysis and the p+1 loop can interact with the substrate. Subdomain IX contains mostly the hydrophobic αF-helix and aspartate, while subdomains X and XI contain α-helices involved in binding substrate proteins [42,141,142]. The so-called D-recruiting site (DRS) is also important to properly coordinating the binding structure and comprises the docking groove (residues 145 to 169), the ED site (residues 196 to 204), and a common docking motif (D-motif) (residues 359 to 372). Figure 3 illustrates a two-dimensional view of JNK3 structure and important regions linked to either JNK signaling or JNK conformation. 

As previously mentioned, the CMGC kinase group shares many similarities within their kinase domain, especially at the catalytic subunit, which catalyzes the transfer of the γ-phosphate from ATP to serine or threonine residues of protein substrates [35]. These identical or very similar domains make it a challenging task to develop selective inhibitors. Potential kinase inhibitors may fail in clinical trials because they bind to multiple targets due to structural similarity. In this case, a deeper understanding of the kinase target of each drug is required to develop successful treatment strategies, although some off-target effects are beneficial once drug repurposing is particularly interesting [143].

**Figure 4:**
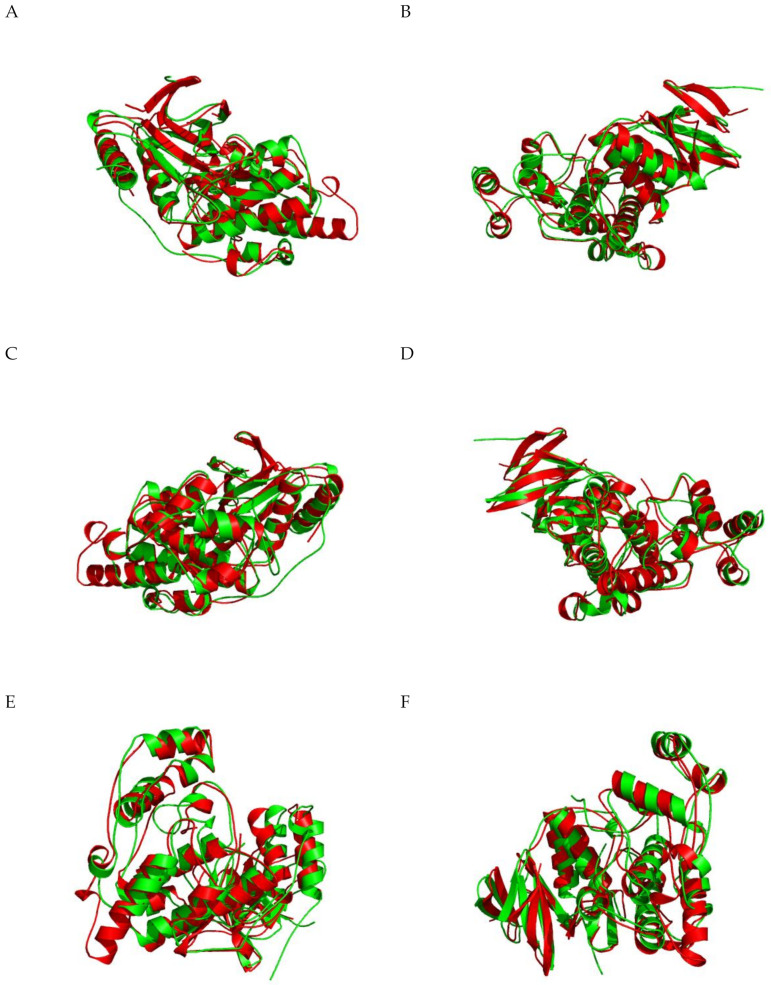

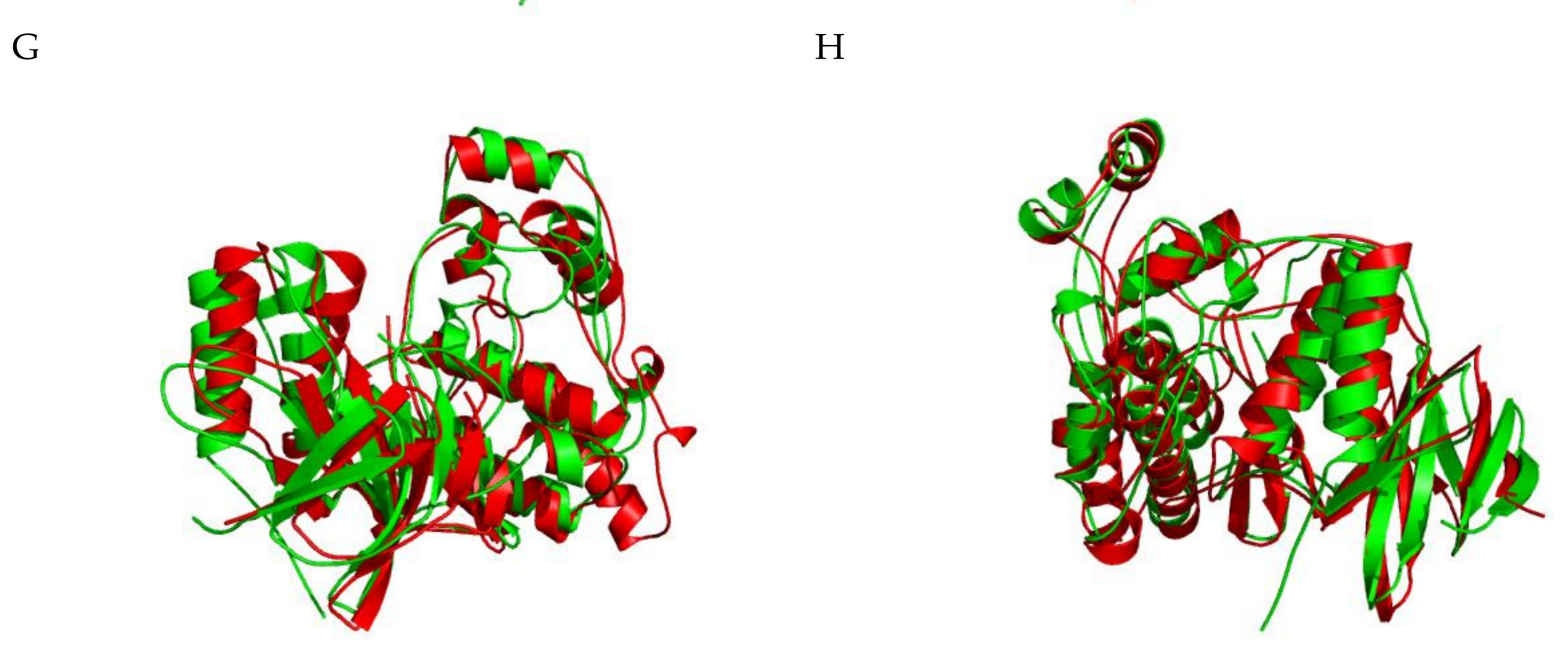
(**A**–**D**) Structural superposition of JNK3 (PDB code: 3OY1, chain A, represented in red) and ERK1 (PDB code: 2ZOQ, chain A, represented in green); (**E**–**H**) Structural superposition of JNK3 (PDB code: 3OY1, chain A, represented in red) and p38α (PDB code: 1A9U, chain A, represented in green). Figure generated by iPBA.

Regarding structure, the homologous region of JNK3 (Phe48-Glu397) is similar to other MAPK (45% identical in amino acid sequence to ERK2 and 51% identical to p38). However, some segments, such as the A-loop region, show a high structure variance between JNK, p38, and ERK. In JNK3, the activation loop is 10 amino acids long, so the A-loop of JNK3 is four residues shorter than ERK and two residues longer than p38. Additionally, the residue between two phosphorylation sites at the Thr-X-Tyr motif is proline in JNK3 (Thr221-Pro222-Tyr223), and therefore, for JNK, the Thr-X-Tyr motif is also called TPY motif. For p38, glycine is present, whereas in ERK it is glutamate. ERK and p38 lack a 12 amino acid sequence at the C-terminal domain that is present in all JNK isoforms’ (residues 283-328). These variations cause changes in the protein structure and impact the drug specificity. Figure 4 (A–D) shows the superposition of JNK3 (represented in red) and ERK1 (represented in green), while Figure 4 (E–H) presents a superposition of JNK3 (red) and p38 (green). 

On the other hand, JNK has three isoforms and several studies already pointed out that it is particularly challenging to design highly selective JNK3 inhibitors because it shares 77% amino acid sequence identity with JNK2 and 75% identity with JNK1 as well. The sequence identity at the ATP binding pocket is 98% identical between all three JNK isoforms. The ATP binding region has been explored as a potential target, but since this is a highly conserved region, drugs targeting the ATP binding pocket lack specificity. On the other hand, the hydrophobic region is an interesting ‘place’ to explore JNK selectiveness but is ‘protected’ by a gatekeeping amino acid residue. The open conformation of the gatekeeper Met146 also contributes to selectivity.

The presence of a leucine residue (Leu144) within the binding pocket could explain the selectiveness of JNK2/JNK3 isoforms over JNK1, which presents an isoleucine residue (Ile106) in the same position. The presence of Leu144 provides the proper accommodation of the naphthalene ring into the selectivity binding pocket, which is not possible in other MAPKs. Another study confirmed this finding when it suggested that Leu144 located within the hydrophobic pocket of JNK3 was responsible for the selectivity [144]. On the other hand, the selectivity of JNK3 over JNK2 is explained by the presence of isoleucine and methionine residues (Ile92 and Met115) in the JNK3 isoform, while the JNK2 isoform presents valine and leucine residues in the same position (Val54 and Leu77). A recent study suggested that hydrophobic interactions with other amino acids, such as valine and glycine, are also necessary to confer a selective inhibition of JNK3 [144]. 

**Figure 5:**
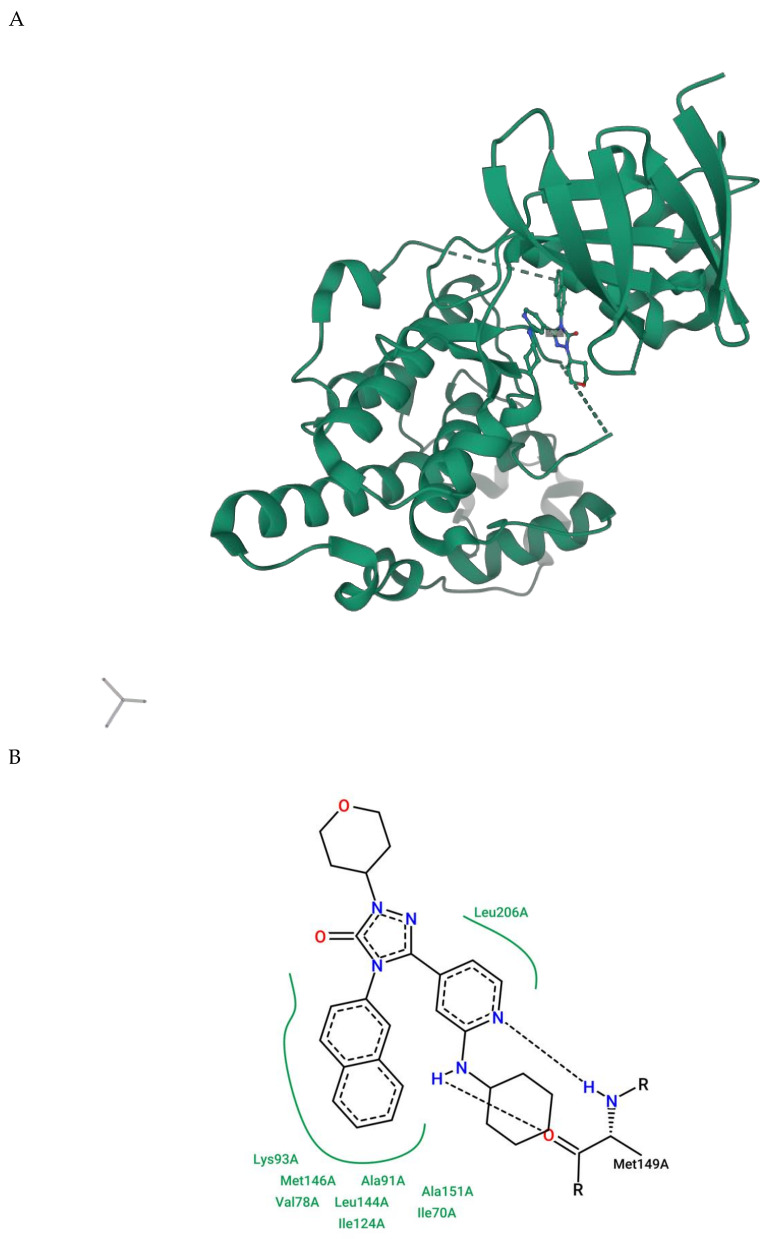
(**A**) Overview of the three-dimensional structure of the JNK3 interacting with its ligand (PDB code: 3OY1); (**B**) 2D protein-ligand interaction plot for 3OY1, emphasizing the interactions between the ligand and the residue Met149. The hydrogen bond (at atom level) is shown in dashed lines. Amino acids that do not interact with the ligand via hydrogen bond are shown in green. Water molecules are not shown. Plot automatically generated by ProteinsPlus using the PoseView tool based on the 2Ddraw library.

Crystallographic structures solved by X-ray of the JNK3 protein are useful for understanding the interaction between ligand and target. The three-dimensional structure of JNK3 bound to an inhibitor with in vitro CNS-like pharmacokinetic properties is shown in Figure 5A. A more detailed interaction between the ligand and JNK3 amino acids is represented in Figure 5B. Experimental studies determined that the molecule 5-[2-(cyclohexylamine)pyridin-4-yl]-4-naphthalene-2-yl-2-(tetrahydro-2H-pyran-4-yl)-2,4-dihydro-3H-1,2,4-triazol-3-one (referred to as 589) preferably inhibits JNK3 (IC50 = 0.016 µM) and JNK2 (IC50 = 0.097 µM) over JNK1 [145].

Despite the ligand appearing to be highly selective to JNK2/3, the ligand molecule was designed to possibly treat AD, and therefore, it must show high biological activity and low toxicity. Authors demonstrated that early ADMET (absorption, distribution, metabolism, excretion, and toxicity) profiling in drug discovery drastically decreases the fraction of pharmacokinetics-related failure in clinical trials [146,147]. In this case, in silico methods can predict the ADMET profiling from a molecular structure, saving time and resources. Another important aspect is that approximately 98% of small organic compounds do not cross the blood–brain barrier (BBB), which is fundamental when dealing with drugs supposed to act at the SNC. This is a crucial issue and must be taken into account in this case.

## 3. Therapeutic Proposal

In 1974, Drachman and Leavitt suggested that memory was related to the cholinergic system and depended on age [148], a notion that is still considered valid today. At the same time, two British groups independently demonstrated that the pathology of AD was associated with a severe loss of central cholinergic neurons, more precisely, the severity of dementia was correlated with the extent of cholinergic loss in the basal nucleus of Meynert [149,150]. The cholinergic hypothesis led to the development of drugs during the 1980s and 1990s and continues to provide a basis for current development efforts with modulators of neuronal nicotinic receptors and other molecules that have effects on cholinergic function such as muscarinic and nicotinic agonists, partial agonists, and allosteric modulators of 5-hydroxytryptamine (5-HT) receptor [148,151,152,153]. So, although other therapeutic targets are being investigated, the cholinergic hypothesis and the amyloid cascade hypothesis have influenced drug development more profoundly, especially by the latter. The amyloid cascade hypothesis has dominated drug development in the past two decades and presents several targets, including inhibition of protein kinase activation [152]. In fact, among the different hypotheses for AD, like amyloid hypothesis, neurotransmitter hypothesis, tau propagation, mitochondrial cascade, neurovascular, exercise, inflammatory, and virus, the first cited was the most strongly tested one in clinical trials until 2019 (22.3%), followed by the neurotransmitter hypothesis, with 19.0% of trials [31].

Despite having been a known disease for over a hundred years, the current treatment of AD seems to be more palliative by restricting itself to the management of symptoms by temporarily delaying cognitive impairment [4,49,50,51,52,53,54]. However, there are still many points that remain uncertain concerning the pathophysiology and the treatment of AD, impacting the development of new drugs. There are currently four drugs approved by the FDA and used clinically, totaling five therapies, the fifth being a combination of two drugs. Clinical treatments are mainly divided into two categories: Acetylcholinesterase inhibitors (AChEIs), represented by donepezil, and N-methyl-D-aspartic acid (NMDA) receptor antagonist, represented by memantine [93]. Unfortunately, only the symptoms are treated because the drugs are regulators of neurotransmitters with no significant effect on the progression of AD. Therefore, it is necessary to seek alternative strategies to treat AD given the limited progress of therapy in phase III clinical trials and the fact that no new drugs have been approved since 2003 [49,55]. About 200 drugs are in phase II of analysis, albeit they show a limited clinical effect, and there is a controversy regarding their therapeutic efficacy. There are many potential drug targets, and so far, there are no validated targets, except perhaps for the cholinergic system [53,55,56]. The ‘normal’ JNK pathway is essential for neurodevelopment and neuronal regeneration, albeit excessive activation of this route can induce apoptosis in neurons. Thus, techniques that allow specific inhibition of JNK have been developed and identified as neuroprotective agents, which can not only treat AD but also other pathologies external to the CNS [44,61,81,154,155,156]. Evidence shows that JNKs can be therapeutic targets in several conditions [157], including Parkinson’s disease [158] and AD [159], obesity and insulin resistance [160,161], rheumatoid arthritis [162], asthma [163,164,165], vascular disease, and atherosclerosis [166]. Given its performance in various biological and pathological processes, different approaches are used to pharmacologically block JNK activity.

In the human body, there are hundreds of kinases, and several of them are somehow involved in different conditions, including AD, and therefore, they constitute an interesting molecular target [167]. The first small molecule kinase inhibitor, Imatinib (Gleevec, Novartis), was FDA-approved in 2001 to treat leukemia. As of June 2020, there are now 61 FDA-approved kinase inhibitors and a simple search for the term ‘kinase inhibitor’ on the ClinicalTrials.gov website revealed 6464 trials, reinforcing its relevance. Most kinase inhibitors comprehend the ATP-competitive inhibitor types. Within this group, type I inhibitors bind to the active DGF-in conformation of the kinase, while type II inhibitors bind to the inactive conformation of the kinase (DFG-out) in a such way that the A-loop blocks the substrate from binding to the enzyme [140,168,169,170,171]. These compounds have a heterocyclic ring that mimics the interaction of the purine ring of the ATP and occupies the adenine binding region of the ATP-binding pocket, while other parts of the molecule occupy adjacent hydrophobic regions [168]. Compared to type I, type II inhibitors have more selectivity as there is higher heterogeneity among inactive conformation states but less affinity to active kinases [172,173]. Aminopyrazoles, aminopyridines, aminopyrimidines, indazoles, pyridine carboxamides, benzothien-2-ylamides, and benzothiazol-2-yl acetonitriles are some small molecules already reported as ATP-competitor JNK inhibitors [157,174].

One of the first molecules to be explored was SP600125 (PubChem ID 8515), a pan-JNK inhibitor with IC50 values for JNK1, JNK2, and JNK3 of 40, 40, and 90 nM, respectively. In vivo and in vitro models of AD demonstrated that SP600125 prevents neuronal death induced by βAPP production. Besides, other studies showed that intracerebroventricular injections of SP600125 improved AD-related neurological aspects in animal models. However, such a compound offers limited specificity. SP600125 helped in understanding the role of JNK in many physiological and pathological conditions, and despite still being broadly used in research, the lack of specificity confers variable degrees of responses and toxicity profiles. Along with all three JNK isoforms, it inhibits upstream kinases of the JNK pathway such as MKK4 and MKK7 but also interferes with the signaling of proteins not directly related to the classical JNK pathway, such as SGK, S6K1 (p70 ribosomal protein S6 kinase), AMPK, CDK2, CK1d, and DYRK1A, a total of at least 13 other kinases [175] [176]. Tanzisertib (Celgene) (PubChem ID 11597537) is a pan-JNK inhibitor developed based on the SP600125 structure with reported inhibition of JNK3 activity (PBD ID 3TTI) [177]. This molecule, also known as CC-930, was the first orally active molecule discovered and was being investigated in phase II clinical trials for treating idiopathic pulmonary fibrosis (NCT01203943) and discoid lupus erythematosus (NCT01466725). Trials were discontinued in 2012 due to increased hepatotoxicity [178,179]. Bentamapimod (also known as AS602801 or PGL5001, PubChem ID 10195250) is another orally active pan-JNK inhibitor (PregLem SA). This ATP-competitive molecule with an IC50 of 80 nM, 90 nM, and 230 nM for JNK1, JNK2, and JNK3, respectively, demonstrated a sufficient safety and toxicity profile in phase I and II clinical trials for inflammatory endometriosis (NCT01630252) [180,181]. In combination with enzalutamide, treatment with Bentamapimod showed antineoplasic activity in a prostate cancer model in vitro [182]. CC-401 (PubChem ID 10430360), developed by Celgene, is a pan-JNK inhibitor derivate from SP600125 with an inhibition constant (Ki) ranging from 25 to 50 nM with 40-fold selectivity for JNK than other kinases. The compound entered a phase I clinical trial (NCT00126893) to determine the optimal dosing for individuals with high-risk myeloid leukemia, but the trial was discontinued [183,184]. However, type I and type II inhibitors bind to the same ATP-binding site, which is a highly conserved domain within the MAPK family [33]. In this case, the design of a specific kinase inhibitor capable of blocking a single kinase by either type I or type II mechanisms of action is a considerable challenge. Since lack of specificity is a disadvantage, non-ATP-competitive inhibitors are thought to be more selective than ATP-competitive inhibitors. While type III inhibitors bind to catalytic sites close to the ATP-binding site, type IV binds to a catalytic site distant from the ATP-pocket. Similarly, in primary cell cultures, compounds such as K252a and CEP1347 inhibited Aβ-induced neuronal death, the latter even reaching the stage of clinical studies. However, it did not perform satisfactorily enough to proceed to the following tests [23,27,36,61,66,67,68,70,90,92,156,185,186,187,188,189,190,191,192]

**Figure 6:**
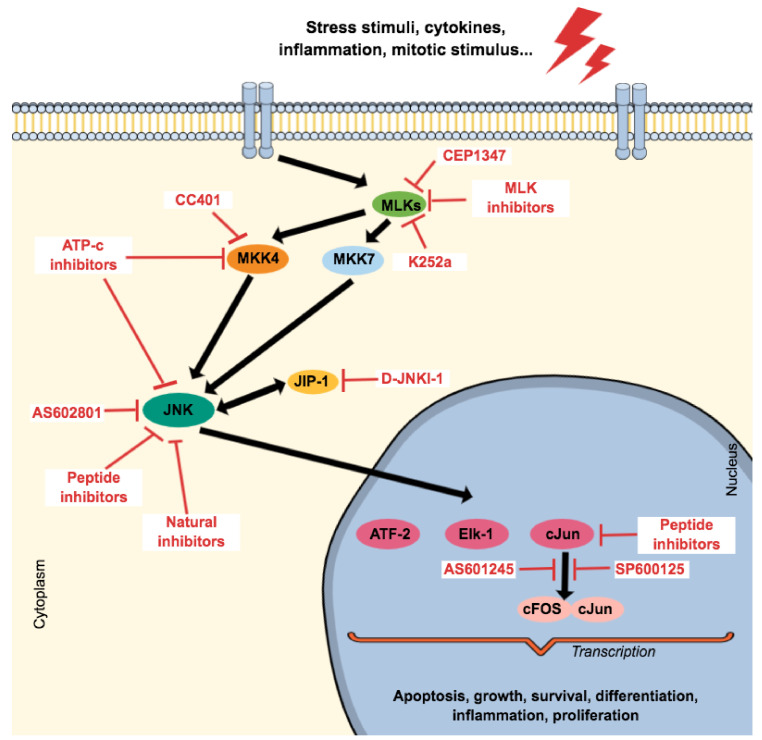
Schematic representation of the mechanism of action of JNK inhibitors.

Type IV kinase inhibitors do not target allosteric regions outside the ATP-binding site or the catalytic site. Instead, they inhibit the enzymatic activity by either blocking the access to upstream activators or by preventing the phosphorylation of some downstream substrates while preserving the biological function of other important substrates. They target allosteric sites at the C-lobe, N-lobe, or allosteric pockets at the kinase domain superficies [193]. Type IV JNK inhibitors disrupt the interaction between JIP-1 and β-arrestin-2 with JNK, which is essential for the function and regulation of JNK signaling, providing critical tools for manipulating the ‘signalosome’ and the cellular response to different stimuli [183]. In this sense, blocking JIP1-JNK could be an alternative to treat neurological diseases because even though knockout of JIP-1 did not affect APP transport or Aβ production [194], it interacts with the APP intracellular domain [195]. Still, JIP-1 inhibition prevented apoptosis in sympathetic neurons [196], and transgenic mice with less JIP-1-mediated JNK activation showed enhanced long-term synaptic plasticity, which is linked to learning and memory skills [197]. In this sense, brimapitide (also known as XG-102, AM-111, or D-JNKI1, PubChem ID 315661186) (Auris Medical AG/Xigen SA) is a cell-permeable peptide and comprises 20 amino acids of the JNK-binding domain JIP-1/IB1 (JBD_20_) conjugated to a carrier peptide derived from HIV-TAT_48-51_ [176] and is a very strong and selective inhibitor in JNKs, and differs from all common chemical inhibitors, inhibiting JNK1, JNK2, and JNK3 with Ki values of 3.3 M, 430 nM and 540nM [183]. In this sense, it is a peptide that inhibits the JNK-JIP interaction (type IV JNK inhibitor) with a reported anti-apoptotic effect in cochlear neurons [198] that was also evaluated in labyrinthitis [199] and acute inflammatory bowel disease in vivo models [200]. The efficacy of intratympanic administration of this agent in the treatment of severe idiopathic sudden sensorineural hearing loss underwent phase II (NCT00802425) and III clinical trials (EudraCT 2013-002077-21/NCT02561091 and NCT02809118) revealing promising results [201,202]. Phase I (NCT01570205) aimed to determine the safety, tolerability, and pharmacokinetics of a single intravenous infusion to treat inflammatory conditions, while the clinical efficacy of a subconjunctival injection with a sterile ophthalmic solution containing brimapitide for treatment of ocular inflammation and pain associated with cataract surgery underwent a clinical trial of phase III (NCT02508337 and NCT02235272), revealing a similar anti-inflammatory effect when compared with dexamethasone eye drops [203,204]. Figure 6 shows the mechanism of action of JNK inhibitors under clinical trials.

**Figure 7:**
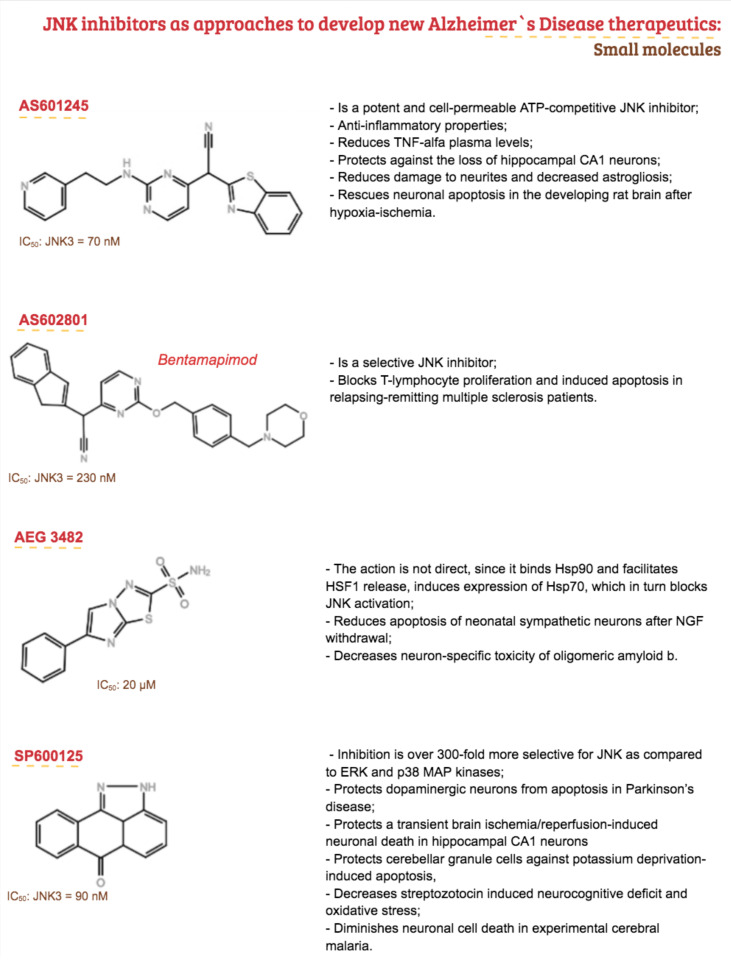

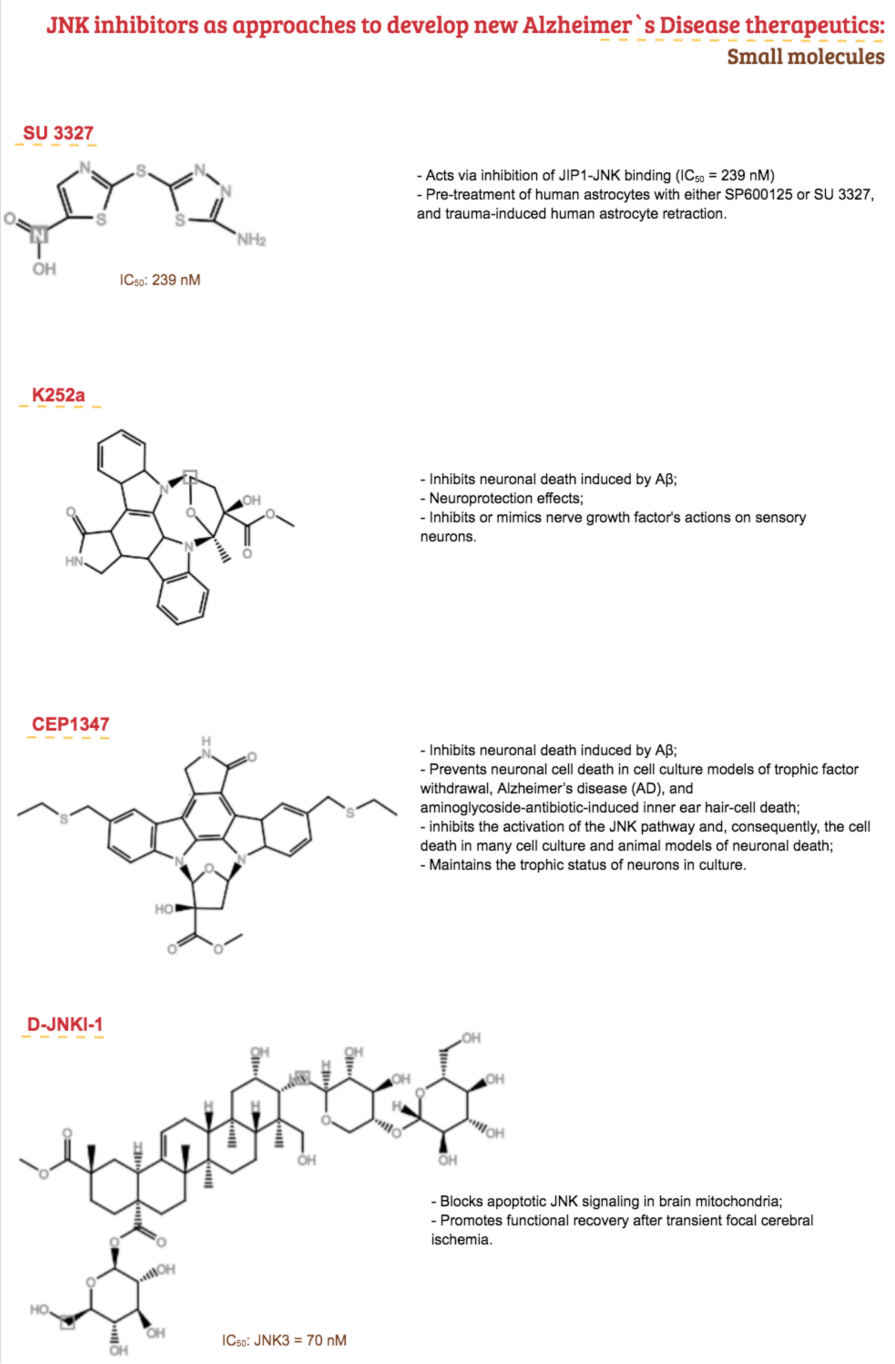
Summary of small molecules of JNK inhibitors used in neurological models that could be used as approaches to develop new Alzheimer’s disease (AD) therapeutics [157,185,186,187,188,189,190,191,192,213,214,215,216,217].

The protective action in diseases of the nervous system has been tested in several experimental models, having the ability to interfere in AD, more specifically with phosphorylation of APP in cortical neurons [183,205,206]. In this sense, it may be a promising drug since it is currently under clinical trials (phase III) but has also shown improved cognitive function, reduced cell death, and pro-inflammatory markers after six months of treatment in an in vivo model of AD [207]. On the other hand, kinase inhibitors that target sites outside the ATP-binding site show low affinity and a dissociation constant (K_D_) in the micro or millimolar range, and this case, the half-life is too rapid (ranging in a microsecond time scale). Additionally, β-arrestin-2, an important scaffolding protein from the JNK3 cascade in the CNS, represents the possibility of developing a specific inhibitor of JNK3 to combat neurodegenerative disorders. Considering that JNK3 is highly expressed in CNS, it is important to note that although β-arrestin-2 can bind to all JNK isoforms through the common binding motif linked to the N terminal of JNK3; it leads to its specific activation without affecting JNK1 and 2 activity. β-arrestin can also contribute to the regulation of synaptic receptors since they are important adapters that link receptors to the clathrin-dependent pathway of internalization [183,208,209]. In addition, type V combines an ATP-competitive ligand to a secondary binding ligand. In this multi-target profile, type V inhibitors have higher potency and selectivity [210]. Molecules that form an irreversible covalent bond with the catalytic site are known as type VI inhibitors [193,211,212]. There are no types III, V, and VI JNK inhibitors under clinical trials. Figure 7 shows the main JNK3 small molecules explored so far with a promising future as therapeutic proposals for AD.

## 4. Conclusions and Perspectives

The heterogeneity of AD, the lack of extensive knowledge about the pathophysiology of AD, and the restricted therapeutic approaches open opportunities for candidate drugs to treat AD in a more effective manner. The high failure rate of the aforementioned test drugs may be because of failing to explore the correct targets.

In this context, JNK3/p38 inhibitors represent an interesting therapeutic alternative since results from post-mortem immunohistochemical studies revealed a significant increase in pJNK and pp38 in the frontal cortex of the brains of patients with AD compared to the brains of control subjects, for example. Already, p38 activates microglia in response to neurotoxic molecules and increases the production of pro-inflammatory cytokines, but shreds of evidence suggest that JNK and p38 are important to AD’s pathophysiology, and the selective p38 inhibitors might not be suitable, especially for elderly and/or anxious individuals. On the other hand, there are some examples of JNK inhibitors tested in vitro and with good results. Despite some molecules being considered not suitable for human use, these inhibitors proved useful in elucidating the role of JNK signaling in neurodegeneration. The key challenge is to improve research in this field and develop orally active molecules that can cross the BBB without generating major side effects.

## Figures and Tables

**Table 1:** c-Jun N-terminal kinase (JNK) isoforms described by Gupta et al. (1996).

JNK1	JNK2	JNK3 ^2^
1α1	2α1	3α1
1α2 ^1^	2α2 ^1^	3α2 ^1^
1β1	2β1	
1β2	2β2	

^1^ Canonical isoform of each JNK. ^2^ A third JNK3 isoform was later described by another research group [77] but is not listed in the present table. This third isoform lacks the first 38 amino acids. Furthermore, according to UniProt database, another 68 potential JNK3 isoforms were mapped in silico [78]. The three JNK3 isoforms are 81.68% identical between each other, while JNK3α1 and JNK3α2 are 89.87% identical.

## Appendix A

**Table A1:** Summary of brain areas and systems commonly affected in AD patients and the major consequences according to Arnold et al. (1991), Firth et al. (2019), and Bruen et al. (2008).

Affected Area/System	Major Consequence
Entorhinal cortex	Memory impairments
Hippocampus	
Medial temporal cortex	
Neocortex	Deficits in higher cognitive functions, such as language, calculation, problem-solving, and judgement
Basal forebrain cholinergic system	Contributes to memory and attention deficits
Limbic cortex	Behavioral and emotional disturbances
Amygdala	
Thalamus	
Monoaminergic system	
